# Use of Di(2-ethylhexyl) Phthalate–Containing Medical Products and Urinary Levels of Mono(2-ethylhexyl) Phthalate in Neonatal Intensive Care Unit Infants

**DOI:** 10.1289/ehp.7932

**Published:** 2005-06-08

**Authors:** Ronald Green, Russ Hauser, Antonia M. Calafat, Jennifer Weuve, Ted Schettler, Steven Ringer, Kenneth Huttner, Howard Hu

**Affiliations:** 1 Department of Environmental Health, Harvard School of Public Health, Boston, Massachusetts, USA; 2 Centers for Disease Control and Prevention, Atlanta, Georgia, USA; 3 Science and Environmental Health Network, Boston, Massachusetts, USA; 4 Neonatology Unit, Brigham and Women’s Hospital, Harvard Medical School, Boston, Massachusetts, USA; 5 Neonatology Unit, Massachusetts General Hospital, Harvard Medical School, Boston, Massachusetts, USA; 6 Channing Laboratory, Department of Medicine, Brigham and Women’s Hospital, Harvard Medical School, Boston, Massachusetts, USA

**Keywords:** di(2-ethylhexyl) phthalate, hospital equipment and supplies, mono(2-ethylhexyl) phthalate, neonatal intensive care units, newborn infants

## Abstract

Objective: Di(2-ethylhexyl) phthalate (DEHP) is a plasticizer used in medical products made with polyvinyl chloride (PVC) plastic and may be toxic to humans. DEHP is lipophilic and binds non-covalently to PVC, allowing it to leach from these products. Medical devices containing DEHP are used extensively in neonatal intensive care units (NICUs). Among neonates in NICUs, we studied exposure to DEHP-containing medical devices in relation to urinary levels of mono(2-ethylhexyl) phthalate (MEHP), a metabolite of DEHP.

Design: We used a cross-sectional design for this study.

Participants: We studied 54 neonates admitted to either of two level III hospital NICUs for at least 3 days between 1 March and 30 April 2003.

Measurements: *A priori*, we classified the infants’ exposures to DEHP based on medical products used: The low-DEHP exposure group included infants receiving primarily bottle and/or gavage feedings; the medium exposure group included infants receiving enteral feedings, intravenous hyperalimentation, and/or nasal continuous positive airway pressure; and the high exposure group included infants receiving umbilical vessel catheterization, endotracheal intubation, intravenous hyperalimentation, and indwelling gavage tube. We measured MEHP in the infants’ urine using automated solid-phase extraction/isotope dilution/high-performance liquid chromatography/ tandem mass spectrometry.

Results: Urinary MEHP levels increased monotonically with DEHP exposure. For the low-, medium-, and high-DEHP exposure groups, median (interquartile range) MEHP levels were 4 (18), 28 (58), and 86 ng/mL (150), respectively (*p* = 0.004). After adjustment for institution and sex, urinary MEHP levels among infants in the high exposure group were 5.1 times those among infants in the low exposure group (*p* = 0.03).

Conclusion: Intensive use of DEHP-containing medical devices in NICU infants results in higher exposure to DEHP as reflected by elevated urinary levels of MEHP.

Di(2-ethylhexyl) phthalate (DEHP) is an aromatic diester used primarily to soften and plasticize the rigid polymer polyvinyl chloride (PVC); DEHP may represent between 20 and 40% of the finished weight of the plastic (Jaeger and Rubin 1973). Among other properties, DEHP imparts to PVC flexibility, strength, optical clarity, and resistance to broad-range temperature variations (Shea 2003). In its pure form, DEHP is a clear, oily liquid, which is highly lipophilic (fat soluble) and poorly soluble in water. In PVC plastic, DEHP resides in the PVC matrix as a semisolid and readily migrates out of the plastic into blood or other lipid-containing solutions in contact with the plastic, a phenomenon observed with blood stored in PVC bags (Peck and Albro 1982; Rock et al. 1984). Because of its versatile properties, DEHP is also found in many commercial and household products such as vinyl floor and wall coverings, furniture, raincoats, and shower curtains, as well as cosmetics, personal care products, and food packaging [Agency for Toxic Substances and Disease Registry (ATSDR) 2002].

DEHP has been used as a plasticizer in a variety of medical products, such as bags containing blood, plasma, intravenous fluids, and total parenteral nutrition, tubing associated with their administration, nasogastric tubes, enteral feeding tubes, umbilical catheters, extra-corporeal membrane oxygenation (ECMO) circuit tubing, hemodialysis tubing, respiratory masks, endotracheal tubes, and examination gloves. The rate of DEHP leaching varies not only with the type of solution in contact with the plastic material but also with storage and temperatures at the time of use, storage time, and percent DEHP in the plastic product (Marcel 1973). The leaching rate of DEHP has been studied most rigorously for banked blood and plasma and, as reported, varies between 0.25 to 0.40 mg/100 mL/day for whole blood stored > 21 days at 4°C and 6 mg/unit of platelet concentrate stored at room temperature (Jaeger and Rubin 1973; Marcel 1973; Peck and Albro 1982). Leaching has also been demonstrated in a study in which endotracheal tubes were found to have 6–12% less (0.06–0.12 mg DEHP/mg tube) DEHP after use (Latini and Avery 1999).

Mono(2-ethylhexyl) phthalate (MEHP), one of the metabolites of DEHP, consistently produces developmental, reproductive, and hepatic toxicity in laboratory animals (ATSDR 2002; Gray and Beamand 1984), raising concern about whether human exposure to DEHP approaches the levels of adverse effect found in toxicologic studies. Medical devices containing DEHP are in extensive use in modern neonatal intensive care units (NICUs). It has been estimated that such use might be exposing infants to DEHP at levels that exceed the average daily adult exposure [median = 0.71 μg/kg body weight/day (Kohn et al. 2000)] by 2–3 orders of magnitude, approaching the lowest observed adverse effect level in animal studies (38–144 mg/kg body weight/day) (National Toxicology Program 2000). A recent study showed that MEHP urinary concentrations in neonates undergoing intensive therapeutic interventions in a NICU were several times higher than those from the general U.S. population (Calafat et al. 2004). In addition to potentially high neonatal exposure in NICUs from DEHP-containing products, there is additional concern regarding potential health risks because, until 3 months of age, infants have immature glucuronidation pathways. Because glucuronidation, via enzymes such as UDP-glucuronosyltransferase, facilitates urinary excretion of phthalates and other xenobiotics, a reduced potential for glucuronidation may lead to slower MEHP excretion and higher levels of MEHP in neonates than in older children and adults (Leeder and Kearns 1997).

Among neonates in NICUs, we conducted a study to characterize DEHP exposure and to quantitatively relate the use of DEHP-containing products to urinary levels of MEHP, a biologic marker of DEHP exposure.

## Materials and Methods

### Study population.

We studied a convenience sample of 54 infants enrolled from the level III NICUs at two major Boston-area hospitals. Level III NICUs provide all newborn care, including mechanical ventilation, high-frequency ventilation, surgery, and cardiac catheterization. We selected infants to reflect a range of diagnoses (including congenital anomalies and developmental and metabolic abnormalities) and NICU care requirements (including ventilation, enteral feedings, parenteral nutrition, and indwelling catheterization). Infants must have been in the NICU at least 3 consecutive days before enrollment, have had a corrected gestational age of ≤44 weeks, and have been born at or transferred to either hospital between 1 March and 30 April 2003. Concerns regarding the sensitivity of this research led to the design and implementation of a data and sample collection protocol that was based on visual inspection and that did not include inspection of the medical records. The study protocol and methods were approved by the institutional review boards of Harvard School of Public Health, Brigham and Women’s Hospital, and Massachusetts General Hospital.

### Assessment of DEHP exposure.

Before data collection, we defined three DEHP exposure categories (low, medium, and high) based on a review of medical products typically used in both NICUs and information provided by their manufacturers with respect to DEHP content. Infants classified as having low DEHP exposure were those receiving primarily bottle and/or gavage feedings. The medium-DEHP exposure group included infants receiving enteral feedings by indwelling gavage tubes either continuously or by bolus feedings; intravenous hyperalimentation by indwelling percutaneous intravenous central catheter (PICC) line, broviac, or umbilical vessel catheter (UVC); and/or nasal continuous positive airway pressure by nasal prongs. The high-DEHP exposure group included infants receiving continuous indwelling UVC, endotracheal intubation, intravenous hyper-alimentation by the central venous route (i.e., PICC line, broviac, UVC), and an indwelling gavage tube (for gastric decompression).

One of the study investigators (R.G.) observed the care of each infant for 1–4 hr per day for 1–3 days per infant, for a total of 3–12 hr of observation per infant. Observations were made during a single day for 37 infants, on 2 days for 12 infants, and on 3 days for 5 infants. During the observations, the investigator took an inventory of products in use for the care of each infant. This information was used to categorize infants into low-, medium-, and high-DEHP exposure groups. None of the infants changed DEHP exposure groups over the course of observation.

### Assessment of urinary MEHP.

The observing investigator collected spot urine samples at the end of each infant’s observation period(s). A total of 81 urine samples were collected from 54 infants. Two or more urine samples came from 17 infants, and of these, four samples were collected concurrently with the first samples, and 18 others were collected 6–72 hr after the first samples. We used these repeated samples to assess intraindividual variability in urinary MEHP levels.

Urine samples were collected by either squeezing the urine from a cotton gauze placed in the infant’s diaper at the beginning of the observation period or from the cotton filling of the diaper the infant wore during the period of observation. The cotton gauze or the removed cotton diaper filling was placed into a 3–5 cc polypropylene syringe (Becton-Dickinson, Franklin Lakes, NJ), the plunger was replaced, and the urine was squeezed into a 2- or 4-cc Nunc (Rochester, NY) cryovial. Urine samples were frozen within 4–6 hr at –35°C and shipped on dry ice to the Centers for Disease Control and Prevention (CDC; Atlanta, GA) for analysis.

Urine specimens were analyzed for 10 phthalate monoesters, including three DEHP metabolites [MEHP, mono(2-ethyl-5-hydroxyhexyl) phthalate, and mono(2-ethyl-5-oxohexyl) phthalate], using automated solid-phase extraction/isotope dilution/high-performance liquid chromatography/tandem mass spectrometry at the CDC. Monobutyl phthalate and monobenzyl phthalate were also frequently detected. In this report, we focus on levels of MEHP, because this metabolite has been most studied and is a valid biomarker of DEHP exposure.

The analytical method used for measuring phthalate metabolites in urine has been described in detail previously (Silva et al. 2004). Briefly, the method involved the enzymatic deconjugation of the phthalate metabolites from their glucuronidated form, followed by automated solid-phase extraction. We used reversed-phase high-performance liquid chromatography to separate the phthalate metabolites from other components in the extracted urine. We then quantified the metabolites by isotope dilution/tandem mass spectrometry. Samples, reagent blanks, and quality control (QC) materials were processed identically. QC materials were analyzed along with the study samples to ensure the accuracy and reliability of the data. QC materials with low concentrations (QCL) and high concentrations (QCH) were prepared from a base urine pool, obtained from multiple anonymous donors as described previously (Silva et al. 2004), dispensed in 5-mL aliquots, and stored at –20°C. Each QC material was characterized by repeated measurements, spanned over several weeks, to define the mean concentrations and the 95% and 99% control limits of MEHP. Each analytical run consisted of 50 (5 QCs, 5 blanks, and 40 unknown) samples. The concentrations of the replicate QCH and QCL materials, averaged to obtain one measurement of QCH and QCL for each run, were evaluated using standard statistical probability rules. All MEHP concentrations, reported in nanograms per milliliter of urine, were blank-corrected. However, MEHP is not typically found in the reagent blank samples, and when it is, it is at concentrations near the limit of detection (LOD; 0.87 ng/mL). Specimens with MEHP levels below the LOD were assigned a value of 0.435 ng/mL, the midpoint between 0 and the LOD.

### Assessment of other variables.

We collected data, as available, on gestational age and length of stay in the NICU. However, data on these variables were incomplete for about half of the infants because, as noted, the sample collection was anonymous and not based on review of medical records. The interpretation of these data is likely to be tenuous because of missing data, and we therefore do not present these results.

### Statistical analysis.

We used Fisher’s exact test to evaluate differences in sex and institution by DEHP exposure group. To evaluate the intraindividual variability of urinary MEHP levels, we computed Spearman correlations between repeated urine specimens. For unadjusted comparisons of urinary MEHP levels by sex, institution, and DEHP exposure group, we used the Mann-Whitney Kruskal-Wallis non-parametric test. We used multiple linear regression to compare urinary MEHP levels across DEHP exposure groups, adjusting for institution and infants’ sex. Because the distribution of urinary MEHP was skewed, we used log-transformed MEHP values in the regression models. In regression models of log-transformed MEHP, the regression parameters estimated MEHP levels in the medium- and high-DEHP exposure groups as a proportion of MEHP levels in the low-DEHP exposure group. Our primary analyses focused on the MEHP levels in the first urine specimen collected from each infant; in secondary analyses, we incorporated first and, where available, second and third specimens, using repeated-measures regression to compare MEHP levels across exposure groups.

We used quantile regression (Koenker and Hallock 2001) to estimate sex- and institution-adjusted quartiles of urinary MEHP for each DEHP exposure group.

## Results

The 54 infants in our study were roughly equally distributed between the two institutions, and 34 (63%) were female (Table 1). Thirteen (24%) infants had low exposure to DEHP-containing products, 24 (44%) had medium exposure, and 17 (32%) had high exposure. Among the first set of urine samples collected from these infants, 11 (20%) had MEHP levels that were less than the LOD (0.87 ng/mL), and the maximum level was 758 ng/mL (geometric mean = 14 ng/mL; geometric SD = 8.6 ng/mL).

### Correlation between urinary MEHP levels in repeated urine samples.

Among the 17 infants with multiple urine samples, urinary MEHP measurements from the same infants were highly correlated. Spearman correlations were 0.9 (*p* < 0.0001; *n* = 17) for urinary MEHP levels in the first and second urine specimens, 0.9 (*p* = 0.04; *n* = 5) for the second and third specimens, and 0.7 (*p* = 0.2; *n* = 5) for the first and third specimens. Although our data were limited, we observed no apparent dissimilarity in the correlation between specimens collected at the same time (correlation coefficient *r* = 0.80; *n* = 4) versus those collected at different times (*r* = 0.88; *n* = 13 first and second samples only).

### Correlates of DEHP exposure group and urinary MEHP.

Compared with infants in the two lowest DEHP exposure groups, infants in the high-DEHP exposure group were more likely to be patients at institution B (Table 1). In unadjusted comparisons, urinary MEHP levels were significantly higher among infants at institution B (Mann-Whitney Kruskal-Wallis *p* = 0.002; Table 2).

### Association of DEHP exposure group with urinary MEHP.

**Figure 1:**
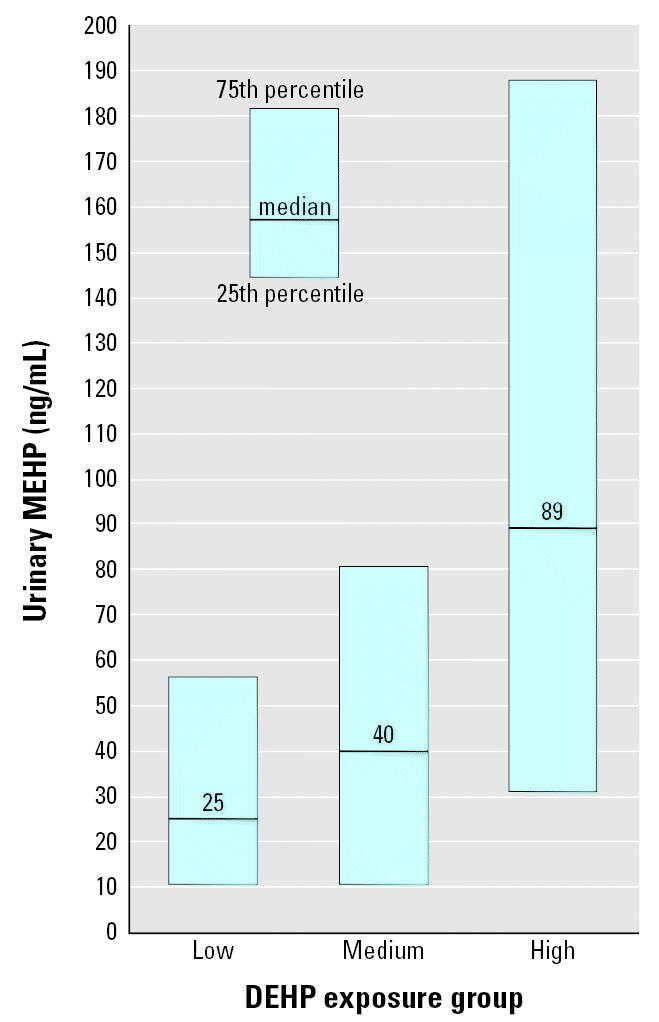
Median and interquartile range of urinary MEHP, by DEHP exposure group, and adjusted for institution and infant sex using quantile regression.

Progressively higher DEHP exposure group was associated with progressively higher urinary MEHP levels. Median urinary MEHP levels (and 25th and 75th percentiles) in infants in the low-, medium-, and high-DEHP exposure groups were 4 (< LOD, 18), 28 (3, 61), and 86 (21, 171) ng/mL, respectively. DEHP exposure group remained a substantial predictor of urinary MEHP levels even after adjusting for infants’ sex and institution of hospitalization (*p* = 0.09; Table 3, Figure 1). Compared with infants in the low-DEHP exposure group, infants in the medium-DEHP exposure group had urinary MEHP levels that were twice as high [95% confidence interval (CI) of the multiplication factor, 0.5–7.4; *p* = 0.3], and infants in the high-DEHP exposure group had levels that were 5.1 times as high (95% CI of the multiplication factor, 1.2–21.9; *p* = 0.03). These results were identical when we analyzed all first, second, and third urinary MEHP values together using repeated-measures regression.

Urinary creatinine levels were available for 67 samples, 45 infants in total. Using this restricted sample, we analyzed the association between DEHP exposure group and creatinine-adjusted urinary MEHP concentrations, and the results were nearly identical to the results from the analyses of unadjusted MEHP levels.

## Discussion

Our study of 54 infants receiving care in two NICUs demonstrated that the use of DEHP-containing medical devices is associated with a monotonic increase in urinary levels of MEHP, a metabolite of DEHP. In particular, urinary MEHP levels among infants in the high-DEHP exposure group were five times as high as those in the low-DEHP exposure group, after adjustment for infants’ sex and institution of care. Urinary MEHP levels among infants in the medium-DEHP exposure group were twice as high as levels among infants in the low-DEHP exposure group. Adjustment for institution resulted in modest attenuation in the association between DEHP exposure group and urinary MEHP, which may reflect the potential influence of other unmeasured sources of DEHP exposure that differed between institutions. In addition, urinary MEHP levels were modestly higher among male infants. Although the length of observation varied across infants, because of the acuteness of care requirements and the indwelling (and therefore continuous) nature of many of the products used, potential DEHP exposure from medical devices in use was stable over each infant’s observation period(s).

To date, there are limited measurements on human neonatal exposures to phthalates (Calafat et al. 2004) and, to our knowledge, no data on whether such exposures are associated with adverse health effects in these neonates. Limited data describe the effects of neonatal exposures on health later in life. A study of 18 adolescents 14–16 years of age who had undergone ECMO as neonates found no apparent abnormalities in their growth and pubertal maturity, and the levels of luteinizing hormone, follicle-stimulating hormone, testosterone (in boys), and estradiol (in girls) were within the normal reference ranges for stage of pubertal development (Rais-Bahrami et al. 2004). It is difficult to draw definitive conclusions from this study, because it was very small, and normal reference ranges for reproductive hormones, physical growth, and sexual maturation are quite wide. Furthermore, the postnatal levels of DEHP or its metabolites at the time of the ECMO treatment were not known.

Our study is limited by the narrow range of descriptive data available on the infants and the small number of infants examined. Yet it is the first study to directly examine the relative use of DEHP-containing medical products among NICU infants in relation to urinary MEHP concentration. Because our study included infants with a range of diagnoses and NICU care requirements, the results of this research may be generalizable to infants of other level III NICUs.

Several studies have considered DEHP exposure or sources of DEHP exposure in NICU infants (Calafat et al. 2004; Karle et al. 1997; Latini and Avery 1999; Loff et al. 2000). Calafat et al. (2004) considered exposure to DEHP from medical procedures in six newborn infants of a university hospital NICU undergoing intravenous infusion for more than 2 weeks. The geometric mean level of MEHP (100 ng/mL) in 33 urine samples collected from these children was several times higher than that from the general U.S. population ≥6 years of age (geometric mean, 3.43 ng/mL) (CDC 2003). The urinary MEHP levels reported in that study were similar to those in the high-DEHP exposure group in our study. Brock et al. (2002) studied 19 toddlers 12–17 months of age in Imperial County, California. Twelve children provided two urine samples, and seven children provided a single sample. Eight urine samples from six children had detectable levels of MEHP. The mean urinary MEHP level was 4.6 ng/mL (SD, 6.4 ng/mL; range, LOD < 1.2 to 47.3). These levels were an order of magnitude lower than the mean in the present study on neonates.

Others have directly assessed sources of DEHP exposure from medical devices among infants. Among neonates, Karle et al. (1997) found that DEHP leached from ECMO circuit tubing at 10.5–34.9 mg/kg, depending on length, type, and size of PVC tubing. Interestingly, circuits with heparin coating did not leach DEHP. Latini and Avery (1999) directly observed the degradation of 44 indwelling PVC endotracheal tubes that had been used in NICU infants for as little as 12 hr. These tubes demonstrated a substantial loss of DEHP, 0.06–0.12 mg DEHP per mg of sample (6–12%), compared with unused tubes.

In our study, DEHP exposure differed significantly by institution. Specifically, most high-DEHP-exposure infants were from institution B, and infants from institution B had a greater median urinary MEHP concentration than did infants from institution A. Loff et al. (2000) studied DEHP leaching from parenteral nutrition infusion lines used for 24-hr fat emulsion administration in 2-kg neonates and found that 25 mL of standard 20% lipid emulsion administered by pump via polyethylene syringes and PVC infusion line yielded samples (after 24 hr at 27°C) having postinfusion DEHP concentrations of 422.8 μg/mL. The authors concluded that the total amount of DEHP for a 2-kg baby caused by lipid emulsions in a day, under these conditions, is approximately 10.2 mg. In our study, we speculate that the higher exposure to DEHP in institution B compared with institution A reflects the extensive use in particular of two DEHP-containing items at institution B: unsiliconized PVC indwelling endotracheal tubes, and a PVC indwelling hemodynamic monitoring UVC used for, among other things, parenteral nutrition. These DEHP-containing medical devices were sparingly used in institution A.

In conclusion, our study demonstrated an association between the use of DEHP-containing medical products and urinary levels of MEHP in NICU infants. This study was designed to evaluate the distribution of exposure to DEHP among NICU neonates only. Additional studies, larger and more comprehensive than the present one, with follow-up of the infants, are needed. Such studies would help determine potential health risks, if any, from DEHP exposure among NICU infants.

## Figures and Tables

**Table 1:** Characteristics, by DEHP exposure group [*n* (%)].

		DEHP exposure group			
Factor	No.	Low (*n* = 13)	Medium (*n* = 24)	High (*n* = 17)	*p*-Value^a^
Sex					> 0.9^b^
Female	34	8 (62)	15 (63)	11 (65)	
Male	18	4 (31)	8 (33)	6 (35)	
Missing	2	1 (8)	1 (4)	0 (0)	
Institution					0.01
A	28	11 (85)	12 (50)	5 (29)	
B	26	2 (15)	12 (50)	12 (71)	

a Fisher’s exact test comparing the distribution of sex and institution, respectively, across DEHP exposure group.

b Excludes infants with missing data on sex.

**Table 2:** Median concentrations (and 25th and 75th percentiles) of urinary MEHP, by sex, institution, and DEHP exposure group.

	Urinary MEHP level (ng/mL)			
Factor	25th percentile	Median	75th percentile	*p*-Value^a^
Sex				0.15
Female	3	20	64	
Male	19	39	75	
Institution				0.002
A	< LOD^b^	12	29	
B	18	58	92	
DEHP exposure group				0.001
Low	< LOD^b^	4	18	
Medium	3	28	61	
High	21	86	171	

a Mann-Whitney Kruskal-Wallis nonparametric test to evaluate differences in urinary MEHP distribution.

b LOD = 0.87 ng/mL.

**Table 3:** Relative^a^ urinary MEHP levels (95% CIs), by DEHP exposure group, as a multiple of urinary MEHP in the low-DEHP exposure group (referent = low).

DEHP exposure group	Not adjusted	Adjusted for sex	Adjusted for institution	Adjusted for sex and institution
Low	1.0 (Referent)	1.0 (Referent)	1.0 (Referent)	1.0 (Referent)
Medium	3.4 (0.9–13.4)	3.1 (0.8–11.2)	2.2 (0.6–8.6)	2.0 (0.5–7.4)
High	12.4 (2.9–53.1)	9.7 (2.4–39.5)	6.1 (1.3–28.2)	5.1 (1.2–21.9)
Test of overall differences among groups, *p*-value	0.0004	0.008	0.06	0.09

a Estimates are multiplication factors derived from regression models of log-transformed urinary MEHP. They compare urinary MEHP levels in the medium- and high-DEHP exposure groups with levels in the low-DEHP exposure group. For example, after adjusting for sex and institution, urinary MEHP levels in the high-DEHP exposure group were about 5.1 times as great as those in the low-DEHP exposure group (95% CI, 1.2–21.9 times as great).
